# Cisplatin, Fluorouracil in Bolus Injection, and Leucovorin in First-Line Therapy for Advanced Gastric Cancer as an Alternative to Protocols With Infusional Fluorouracil

**DOI:** 10.1200/JGO.18.00176

**Published:** 2019-01-22

**Authors:** Rafael C. Coelho, Pedro D.P. Abreu, Mariana R. Monteiro, Ana Paula Stramosk, Alvaro Henrique I. Garces, Andreia Cristina Melo, Marcia S. Graudenz, Carlos Jose C. Andrade

**Affiliations:** ^1^ Instituto Nacional de Câncer (INCA), Rio de Janeiro, Brazil; ^2^ Universidade Federal do Rio Grande do Sul (UFRGS), Porto Alegre, Brazil; ^3^ Hospital de Clinicas de Porto Alegre, Porto Alegre, Brazil

## Abstract

**PURPOSE:**

Gastric cancer (GC) is the fourth most common cancer and the second leading cause of cancer death worldwide. Platinum agents and fluoropyrimidines are the main compounds used in the first-line setting for advanced GC. Given the activity of fluorouracil (FU) bolus, the PFL protocol, a chemotherapy regimen combining cisplatin, FU bolus, and leucovorin, was incorporated at the Brazilian National Cancer Institute, because this schedule does not require hospitalization or infusion pumps. This study aims to evaluate the outcomes of PFL in the first-line setting for patients with advanced GC.

**MATERIALS AND METHODS:**

This was a retrospective cohort study evaluating patients with advanced GC treated in the first-line setting with cisplatin 80 mg/m^2^ on day 1 and FU bolus 400 mg/m^2^ plus leucovorin 20 mg/m^2^ on days 1, 8, 15, and 22 every 4 weeks, from January 2008 to December 2014.

**RESULTS:**

A total of 109 patients were enrolled. The median number of cycles received per patient was four (one to 11). Complete responses were achieved in 6.4% and partial responses in 14.7%. Median progression-free survival was 6.3 months (95% CI, 5.08 to 7.58 months) and median overall survival was 8.3 months (95% CI, 6.79 to 9.87 months). Thirty-four (31.2%) patients were alive in 1 year. Grade 3 and 4 adverse events were experienced by 26.6% and 3.7% of patients, respectively, with dose reduction necessary in 9.1%.

**CONCLUSION:**

PFL is active in advanced GC and could be an alternative for FU continuous infusion protocols in institutions with limited resources and/or low budget, which is the reality in many nations all over the world.

## INTRODUCTION

Gastric cancer (GC) is a major health problem, and it is the fourth most common cancer and the second leading cause of cancer death worldwide. More than 950,000 new cases are diagnosed every year. It was estimated that 720,000 patients died as a result of gastric cancer in 2012.^1,2^ In Brazil, 13,540 new cases of GC are expected for men and 7,750 for women in 2018, being the fourth most incident tumor type in men and the sixth in women.^3^

Patients with GC have a poor prognosis, with median survival of around 12 months when treated with cytotoxic chemotherapy with or without target therapies.^4^ First-line treatment of advanced GC is historically based on combinations of platinum compounds and fluoropyrimidines. These drugs can also be associated with taxanes and anthracyclines; for human epidermal growth factor receptor 2–positive tumors, trastuzumab combined with fluoropyrimidine and platinum-based chemotherapy is considered the standard of care.^4^

Fluorouracil (FU) may be administered by intravenous bolus or as a continuous infusion, and each protocol influences its pharmacological behavior and cytotoxic effects involving RNA and DNA synthesis.^5^ FU can be incorporated into nuclear RNA in the form of fluorouridine 5′-triphosphate,^6^ and its impact on DNA synthesis is mainly through the inhibition of thymidylate synthase by 5-fluoro-2′ deoxyuridine-5′ monophosphate^7^ and, to a lesser extent, through its incorporation into DNA.^8^ Sobrero et al^9^ highlighted the role of decreased thymidylate synthase inhibition in the mechanism of resistance to infusional FU, in contrast to the role of decreased incorporation of FU into RNA in the mechanism of resistance to FU bolus.

In vivo pharmacokinetic comparison of bolus versus continuous infusion FU administration shows that the latter results in more constant plasmatic drug levels.^10^ Given that the cytotoxicity of FU is optimal during cell division, the constant drug levels achieved by continuous infusion ensure that a larger number of cells are exposed to FU during the cell cycle.^11^ The superiority of continuous infusion FU administration when compared with bolus infusion in the treatment of metastatic colorectal cancer is highlighted in a meta-analysis of seven randomized studies on the basis of data from 1,219 individual patients.^12^ The continuous infusion resulted in a higher response rate (22% *v* 14%; *P* = .002) and a small but significant survival difference (12.1 *v* 11.3 months; *P* = .039). None of the individual trials included in the meta-analysis had reported a significant survival benefit.^12^ Regarding gastric cancer, there is no trial comparing these two administration modes directly.

**CONTEXT**

Is it possible to treat patients with advanced gastric cancer (GC) with bolus fluorouracil (FU) infusion in the first-line setting?
Treatment with a regimen containing cisplatin, bolus FU, and leucovorin (PFL) was demonstrated to be active and have a favorable toxicity profile in patients treated in the first-line setting of advanced GC.
PFL demonstrated an overall survival of 8.3 months, in line with main phase III randomized trials.
PFL protocol could be an alternative for treatment of advanced GC in institutions with limited resources and/or budget without access to infusion pumps and/or capecitabine, which can be the reality in many nations all over the world.

Given the activity of FU bolus administration shown in many preclinical and clinical studies, similar outcomes when compared with infusional protocols,^5-12^ and the convenient schedule without the need of hospitalization or infusion pumps, PFL protocol, a chemotherapy regimen combining cisplatin (CDDP), FU in bolus infusion, and leucovorin (LV), was incorporated at the Brazilian National Cancer Institute (INCA). This study aims to evaluate the outcomes of the PFL protocol for advanced GC.

## MATERIALS AND METHODS

All patients included in this study were treated, after obtaining their consent, from January 2008 to December 2014 at the INCA (Rio de Janeiro, Brazil). This study was approved by the INCA’s Ethics in Human Research Committee and conducted in accordance with the Declaration of Helsinki and Good Clinical Practice guidelines.

This retrospective cohort study evaluated patients with advanced GC defined as those with metastatic or unresectable disease. Patients were identified through an internal database. Advanced disease had to be documented by imaging and treated in the first-line setting with CDDP 80 mg/m^2^ on day 1; FU 400 mg/m^2^ on days 1, 8, 15, and 22; and LV 20 mg/m^2^ on days 1, 8, 15, and 22 every 4 weeks (PFL regimen). CDDP was diluted in 400 ml of normal saline and mannitol 20% 100 ml, being infused over 60 minutes, followed by LV diluted in 100 ml of 5% glucose solution over 15 minutes and FU in bolus diluted in 100 ml of normal saline after LV. Hydration and electrolytes replacement were performed before and after CDDP infusion.

Clinical data were collected from medical records and included demographics, stage, histology, Eastern Cooperative Oncology Group performance status, clinical and imaging response assessment, tumor characteristics, adverse events, and prior neoadjuvant and adjuvant treatments. Response to treatment was assessed using clinical and radiologic criteria as follows: complete response, partial response, progressive disease (PD), and stable disease (SD). The radiologic evaluation was based on the Response Evaluation Criteria in Solid Tumors, version 1.1,^13^ with a frequency determined by the assistant physician. Evaluation of drug toxicities was standardized according to the National Cancer Institute Common Toxicity Criteria, version 3.0.^14^ Patients receiving the PFL regimen for second-line advanced GC, neoadjuvant and/or adjuvant setting, were excluded, as well as patients with another primary cancer (except nonmelanoma skin cancer) and patients for whom data were not available in medical charts.

Although this study was not designed for a cost-effectiveness analysis, an estimated budget of each of the most common first-line chemotherapeutic regimens per cycle was performed considering only the cost of the drugs, hospitalization, and/or devices. The standard body surface for estimating treatment costs was 1.85 m^2^. The costs of drugs, devices, and procedures are those currently applied to the Brazilian Public Health System. The cost of antiemetic drugs was $5.00 in regimens without CDDP and $7.00 with it. We did not have neurokinin inhibitors at our disposal, which could increase the costs of antiemetic drugs substantially. Protocols with the need for prolonged infusion had the cost of hospital stay per day (US$95.99/d) or infusion pump device (48 hours, US$39.19 and 96 hours, US$39.35) added. Those who received the treatment through an infusion pump had the catheter implantation costs of US$218.18 added to the value described in Table 1. To calculate the costs in US dollars, the conversion rate of US$1.00 = R$3.75 was applied.

**TABLE 1:**
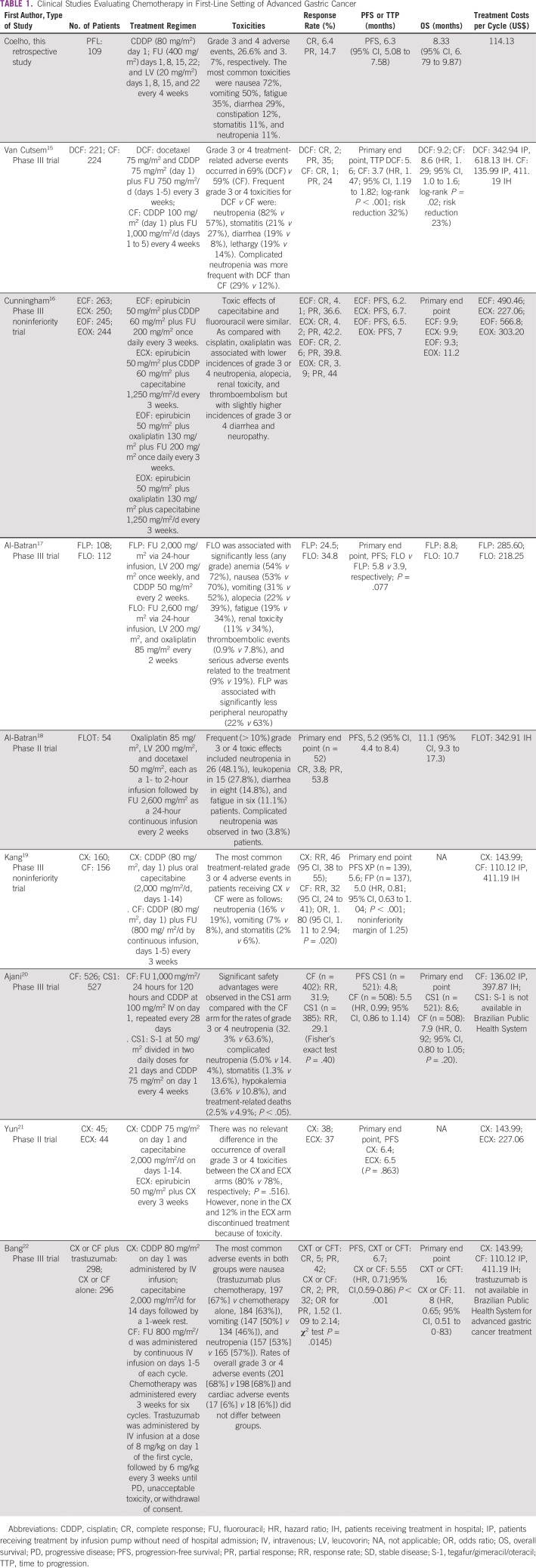
Clinical Studies Evaluating Chemotherapy in First-Line Setting of Advanced Gastric Cancer

Overall survival (OS) was estimated from the time of the first day of infusion of the PFL regimen until death or, for living patients, the last available follow-up. PFS was measured from the starting date of the PFL treatment to either first disease progression or death or the date of the last contact for patients who are alive and progression free, in both cases using the Kaplan-Meier method. All descriptive analyses were performed with SPSS Statistics for Windows, version 18.0 (SPSS, Chicago, IL).

## RESULTS

**FIG 1:**
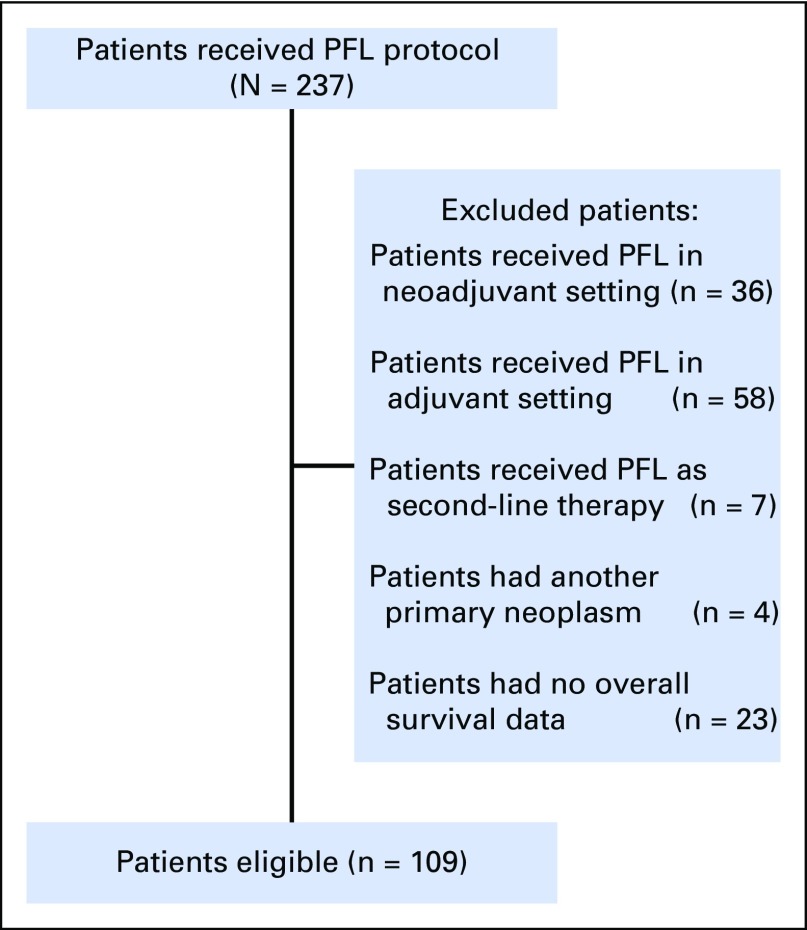
Flowchart of patients eligible for the study. PFL, cisplatin, fluorouracil bolus, and leucovorin.

One hundred nine patients were eligible for this study (Fig 1). Tumor characteristics at diagnosis and epidemiologic data are listed in Table 2.

**TABLE 2:**
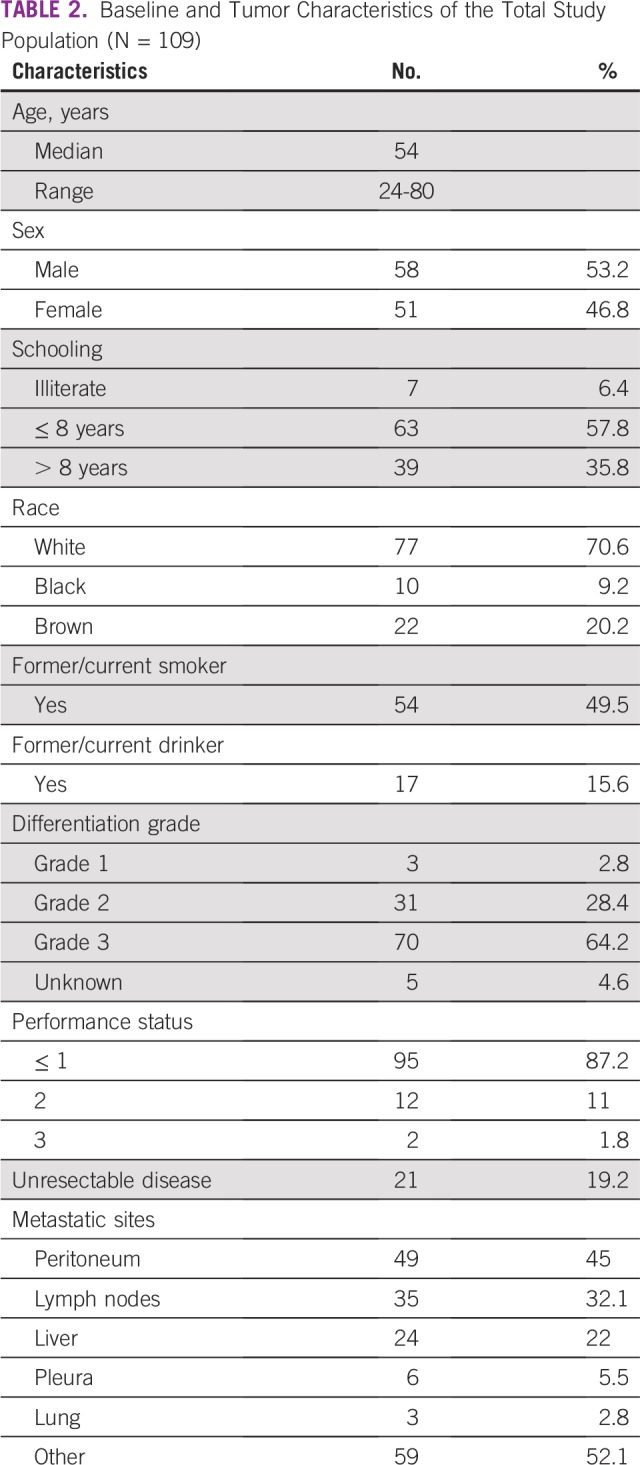
Baseline and Tumor Characteristics of the Total Study Population (N = 109)

The median number of cycles of treatment received per patient was four (one to 11). The median interval between radiologic evaluations was 4 months. Seven patients (6.4%) achieved complete response, 16 (14.7%) had partial response, 16 (14.7%) SD, and 54 (49.5%) PD. Sixteen patients (14.7%) had no response assessment described in their medical records.

**FIG 2:**
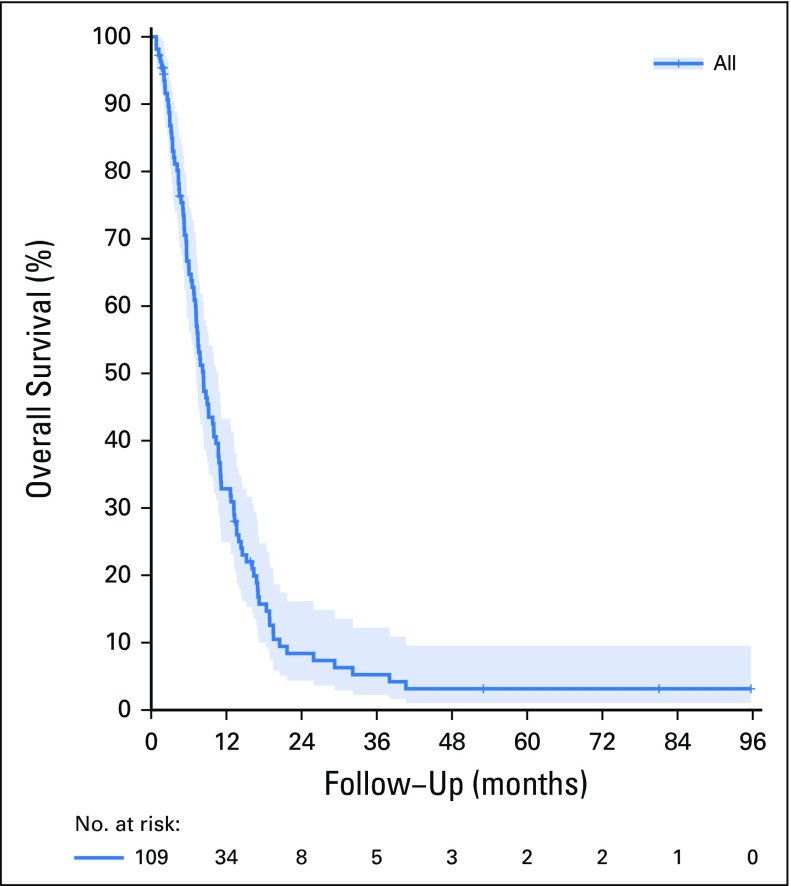
Overall survival among patients with advanced gastric cancer treated with palliative cisplatin, fluorouracil bolus, and leucovorin. Overall survival, 8.3 months (95% CI, 6.79 to 9.87 months).

The median PFS was 6.3 months (95% CI, 5.08 to 7.58 months), and median OS was 8.3 months (95% CI, 6.79 to 9.87 months; Fig 2). Thirty-four (31.2%) patients were alive after 1 year and eight patients (7.3%) after 2 years.

No differences were found in PFS and OS between patients with nonmetastatic and metastatic GC at diagnosis. Table 1 lists the outcomes of the PFL regimen and compares it with important phase II and III clinical trials evaluating the role of platinum compounds and FU in advanced GC.

The most common toxicities in all grades were nausea (72%), vomiting (50%), fatigue (35%), diarrhea (29%), constipation (12%), stomatitis (11%), and neutropenia (11%). Of all adverse events, grade 3 and 4 corresponded to 26.6% and 3.7%, respectively. Three patients had grade 4 neutropenia, one had grade 4 febrile neutropenia, and one died as a result of dehydration from diarrhea. Dose reduction was necessary in 9.1% of cases.

Three patients were re-exposed to the PFL regimen after progressive disease, and only 27 patients (24.8%) received second-line treatment. Three were treated with an oxaliplatin-based regimen and 18 with irinotecan single agent.

## DISCUSSION

GC is a major concern in many countries around the world, especially in developing nations; its burden remains high in Asia, Latin America, and Central and Eastern Europe.^1-3^ Low educational status, negligence, limited access to the public health system, social disparities, scarce resources, high prevalence of smoking and alcoholism, and dietary habits are important factors influencing this epidemiologic data. Unfortunately, treatment of advanced GC is still an unmet need, and new and effective therapeutic strategies are needed across all lines of therapy.^4^

Chemotherapy is a well-established treatment of advanced GC, improving the quality of life and OS.^12,15-20,22,23^ In this study, survival, response, and toxicity were evaluated in a cohort of patients with GC treated with FU bolus and CDDP in the first-line setting.

In an indirect comparison, patients’ outcomes with PFL protocol were similar to those already published in the literature, as shown in Table 1.^15-22^ The OS was 8.3 months (95% CI, 6.79 to 9.87 months) and PFS 6.3 months (95% CI, 5.08 to 7.58 months). Some explanations for the timeframe proximity of these two outcomes are the low number of patients receiving second-line treatment and the absence of standardization for response assessment.

The response rate was also similar to other trials showing 21.1% of patients with tumor shrinkage after treatment. The proportion of PD as best response was greater than what was previously described in the literature,^15-22^ reaching 49.5% of patients; nonetheless, this could be related to the absence of standardization in time of imaging and clinical evaluation for response assessment. Therefore, patients without symptoms had their imaging evaluation postponed; despite the likelihood of having objective response or SD, they were considered as nonresponders, taking into consideration that imaging was performed only during the symptomatic phase of PD. This hypothesis is reinforced in this retrospective study because the main end point, OS, is within the range of the three main phase III trials evaluating the CDDP and FU combination, which is between 7.9 and 8.6 months.^15,20^

Toxicities were manageable, and the treatment with PFL was well tolerated. The most common adverse events were nausea and vomiting, but mainly grades 1 and 2. The explanation for the high prevalence of these two toxicities, besides the high dose of CDDP, is that from 2008 to 2011 there were no neurokinin 1 and 5-HT_3_ antagonists for vomiting prophylaxis available for patients treated in our institution; the latter was introduced routinely only after 2012. Neutropenia occurred in 13.76% of patients. Some other chemotherapy regimens have shown antitumor activity, but their toxicity profiles can limit their use in this usually frail population.^4^ However, PFL was shown to be feasible and a tolerable treatment for this group of patients.

The concern about adverse events is related to the different toxicities between bolus and infusional FU schemes. A meta-analysis revealed that grade 3 and 4 hematologic toxicity (especially neutropenia) was seven times more common in patients who received bolus infusion (*P* < .001). The risks of severe diarrhea, nausea, vomiting, and stomatitis were not different between the two groups. The risk of developing hand-foot syndrome was lower with FU bolus than with FU continuous infusion (13% and 34%, respectively; P < .0001).^12^

In terms of costs per cycle, PFL and combinations with capecitabine may be a cheaper alternative than the majority of other regimens, as described in Table 1. The reduction in costs and time spent in infusion could expand the access to treatment to hospitals with low budget and limited infrastructure.

The retrospective methodology; absence of a strict control in the intervention group; missing data in patient records; lack of standardized chemotherapeutic regimens in neoadjuvant, adjuvant, and second-line settings; and absence of a standardized schedule for imaging response evaluation are the main limitations in this study. On the other hand, the strongest points of this study are that it is a large cohort evaluating a group of patients with advanced GC receiving only one chemotherapeutic regimen, and it shows the treatment of GC in a real-life setting, outside a clinical trial.

The PFL protocol is feasible and well tolerated, and its outcomes are in line with the main prospective phase III trials that evaluated first-line treatment of advanced GC.^15-22^ It is necessary to highlight that the main objective of this study was not to directly compare the outcomes of this retrospective cohort with those clinical trials already published. However, PFL could be an alternative for those institutions that lack the resources to offer the standard-of-care protocols, widening treatment access for patients. In conclusion, a chemotherapy protocol combining CDDP, FU in bolus injection, and LV could be an active and feasible alternative for FU continuous infusion protocols in the outpatient setting for low-budget and resource-limited institutions, which is pragmatically the reality of many nations all over the world.
